# Development of deep pelvic endometriosis following acute haemoperitoneum: a prospective ultrasound study

**DOI:** 10.1093/hropen/hoae036

**Published:** 2024-05-29

**Authors:** Prubpreet Chaggar, Tina Tellum, Lucrezia Viola De Braud, Sarah Annie Solangon, Thulasi Setty, Davor Jurkovic

**Affiliations:** EGA Institute for Women's Health, Faculty of Population and Health Sciences, University College Hospital, London, UK; EGA Institute for Women's Health, Faculty of Population and Health Sciences, University College Hospital, London, UK; Department of Gynaecology, Oslo University Hospital, Oslo, Norway; EGA Institute for Women's Health, Faculty of Population and Health Sciences, University College Hospital, London, UK; EGA Institute for Women's Health, Faculty of Population and Health Sciences, University College Hospital, London, UK; EGA Institute for Women's Health, Faculty of Population and Health Sciences, University College Hospital, London, UK; EGA Institute for Women's Health, Faculty of Population and Health Sciences, University College Hospital, London, UK

**Keywords:** endometriosis, ultrasound, pelvic pain, imaging, adhesions, pathophysiology, haemoperitoneum, quality of life

## Abstract

**STUDY QUESTION:**

Is acute haemoperitoneum that is managed conservatively a precursor of deep endometriosis?

**SUMMARY ANSWER:**

Our study provides evidence to suggest that acute haemoperitoneum may lead to the development of deep endometriosis in a significant proportion of cases.

**WHAT IS KNOWN ALREADY:**

A recent pilot study was the first to suggest that acute haemoperitoneum could be a precursor of deep endometriosis. However, the sample size was small, and the follow-up was not standardized owing to unknown rates of clot absorption and development of endometriosis.

**STUDY DESIGN, SIZE, DURATION:**

This was a prospective observational cohort study conducted at a single centre over a 31-month period. A required sample size of 30 was calculated using results from a previous study, with a minimum of 15 women each in the groups with and without significant haemoperitoneum (study and control groups, respectively). A total of 59 women were recruited to the study and eight were lost to follow-up. The final sample comprised 51 women, 15 in the study group and 36 in the control group.

**PARTICIPANTS/MATERIALS, SETTING, METHODS:**

All non-pregnant, premenopausal women aged 18–50 years who consecutively presented to our dedicated gynaecological diagnostic unit with severe acute lower abdominal pain were eligible for this study. We only included women who were clinically stable and were suitable for conservative management. Those with prior history or evidence of endometriosis on their initial ultrasound scan, previous hysterectomy, or bilateral oophorectomy were excluded. Participants had standardized follow-up visits for 6 months, with pelvic ultrasound scans and the British Society of Gynaecological Endoscopy pelvic pain questionnaires completed at each visit. The primary outcome was the sonographically confirmed presence of newly formed endometriosis. Secondary outcomes were the presence and change of pelvic pain symptoms and health-related quality of life (HR-QOL).

**MAIN RESULTS AND THE ROLE OF CHANCE:**

After completion of follow-up, 7/15 (47%; 95% CI 21.3–71.4%) women presenting with acute haemoperitoneum (study group) developed sonographic evidence of deep endometriosis, compared to 0/36 (0%; 97.5% CI 0.0–9.7%) women in the control group. A ruptured functional haemorrhagic cyst was the most common cause of haemoperitoneum, occurring in 13/15 cases (87%). The time from the initial event to sonographic evidence of endometriosis varied from 2 to 6 months. The EuroQol visual analogue scores were not significantly different at baseline between the groups that developed and did not develop endometriosis [28 (interquartile range (IQR) 15–40, n = 6) vs 56 (IQR 35–75, n = 44), *P *=* *0.09], while the EuroQol-5D values were lower in the endometriosis group [−0.01 (IQR −0.07 to 0.19, n = 6) vs 0.62 (IQR 0.24–0.73, n = 44), *P *=* *0.002]. At 6 months, the EuroQol-5D scores were improved in both groups, but remained significantly lower in the endometriosis group compared to the no endometriosis group [0.69 (IQR 0.66–0.80, n = 6) vs 0.85 (IQR 0.76–1.00, n = 44), *P *=* *0.03]. There was no clinically relevant difference in the pelvic pain scores at either time point.

**LIMITATIONS, REASONS FOR CAUTION:**

It remains uncertain whether minimal, superficial endometriosis existed at commencement of the study and had a role in the development of deep endometriosis. Although the ultrasound findings were in keeping with deep endometriosis, this was not confirmed histologically. The pelvic pain and HR-QOL findings could have been influenced by the baseline scores being taken when the patient was admitted with acute pain. Also, the sample size was too small to draw reliable conclusions regarding the impact of newly developed endometriosis on QoL.

**WIDER IMPLICATIONS OF THE FINDINGS:**

Our study provides further evidence showing that significant haemoperitoneum may be a precursor of deep endometriosis. Haemodynamically stable women presenting with acute pelvic pain and significant haemoperitoneum should be counselled about the risk of developing deep endometriosis. Interventional studies should be carried out in the future to see whether laparoscopy and pelvic washout could prevent development of deep endometriosis. Preventative strategies, including treatment to suppress ovulation and formation of functional cysts, should be further investigated. This includes the combined and progesterone-only contraceptive pills. Larger future studies are also required to assess women over a longer period of time, with adjustment for confounding factors, to evaluate a possible effect on HR-QOL and pain symptoms.

**STUDY FUNDING/COMPETING INTEREST(S):**

Funding was obtained from The Gynaecology Ultrasound Centre, London, UK. TT received personal fees from GE, Samsung, Medtronic, and Merck for lectures on ultrasound. TT also received a postdoctoral grant from the South-Eastern Norwegian Health Authority (grant number 2020083).

**TRIAL REGISTRATION NUMBER:**

researchregistry6472.

WHAT DOES THIS MEAN FOR PATIENTS? Endometriosis is a common condition mainly affecting women of reproductive age. It occurs when tissue similar to the lining of the womb is found elsewhere in the body, most frequently in the organs and structures surrounding the womb. It can cause severe pain symptoms in some women, consequently having significant negative impact on their quality of life.

We still do not fully understand how this condition develops. Several theories have been proposed; however, the cause may not be the same in all cases of endometriosis. A recent study has shown that women who come to casualty with severe pelvic pain and found to have a lot of blood in their pelvis, are more likely to develop deep endometriosis than women without internal bleeding. However, that study was quite small, and we wanted to find out whether we would find the same result by running a larger study. We asked all women to come back for extra scans after discharge from hospital and we followed them up for 6 months. We also asked them to complete pelvic pain and quality of life questionnaires at each visit.

Our results confirmed that almost a half of women who had blood clots in their pelvis developed deep endometriosis. In comparison, none of the women without signs of internal bleeding had signs of endometriosis at follow-up. We concluded that having a significant amount of blood in your pelvic cavity can lead to development of deep endometriosis. We did not find major differences in how much pelvic pain women with and without new endometriosis had. Their quality of life was also similar when they first came to hospital compared to follow-up; however, our study was likely too small to look at the effect of endometriosis on these parameters and we will need to see more women for a longer period of time to answer this question.

## Introduction

Endometriosis is defined as the presence of endometrium-like epithelium and/or stroma outside the uterus, typically associated with an inflammatory process (International Working Group of AAGL, ESGE, ESHRE and WES *et al.*, 2021). This condition remains a ‘hot topic’ in the public eye, with ongoing delays in diagnosis (Ghai *et al.*, 2020) and no recent improvements in the treatment efficacy. It can cause a wide range of pelvic pain symptoms and subfertility (Becker *et al.*, 2022) which has negative impact on women’s quality of life (QOL) (Nnoaham *et al.*, 2011).

Better understanding of the aetiology and pathophysiology of endometriosis is required to develop novel treatments and preventative measures, which are currently lacking. Existing theories include those of retrograde menstruation, coelomic metaplasia, Müllerian remnants, benign metastasis, and the ability of progenitor cells from the bone marrow to differentiate into endometrial tissue (Burney and Giudice, 2012; Zubrzycka *et al.*, 2020; Saunders and Horne, 2021). Development of this disease is, however, likely to be multifactorial.

A recent pilot study observed that acute haemoperitoneum, which is managed conservatively, could be a precursor of deep endometriosis (Bean *et al.*, 2019a). Over time, blood clots became more organized and owing to gravitational effects, settled in dependant parts of the pelvis where the formation of deep endometriotic lesions was subsequently seen on an ultrasound scan. This was the first study to demonstrate the development of deep endometriosis *de novo*, which is an important finding in helping to identify women at risk and target preventative strategies.

The sample size used in this study was, however, small, with only six women with haemoperitoneum who were followed up. Furthermore, there was difficulty in standardizing follow-up for women, as the rates of clot resolution and development of deep endometriosis were unknown when the study was commenced.

In the present study, a larger consecutive cohort of premenopausal, non-pregnant, and haemodynamically stable women attending our gynaecology clinic with severe acute lower abdominal pain were sonographically observed over a standardized period of time. The aim of this study was to determine whether the presence of significant haemoperitoneum in these women would lead to *de novo* development of deep endometriosis and associated symptoms.

## Materials and methods

### Study setting, patient population, and patient flow

This was a single-centre, prospective observational cohort study performed in an acute gynaecology unit between August 2019 and March 2022. All women aged 18–50 years who consecutively presented to our clinic with severe acute lower abdominal pain were eligible for the study. Severe acute lower abdominal pain was described as recent onset pain, resulting in attendance to the emergency department. Further inclusion criteria were being clinically stable, able to tolerate transvaginal ultrasound scan (TVS), and suitable for conservative management.

Exclusion criteria were prior history or sonographic evidence of endometriosis on the initial encounter, previous hysterectomy, bilateral oophorectomy, or postmenopausal status; the latter was described as ≥12 months of amenorrhoea, which was not secondary to breastfeeding, exogenous hormones, or endocrine conditions. We also excluded women who were pregnant, as the presence of significant haemoperitoneum is not suitable for conservative management in this group.

Following the initial assessment, women who met the inclusion criteria were approached about joining the study. Those who agreed were asked to sign a consent form and complete the standardized ‘BSGE pelvic pain questionnaire’ (https://www.bsge.org.uk/history-of-the-endometriosis-centre-project/; Byrne *et al.*, 2018). Clinically stable women with sonographic evidence of significant haemoperitoneum, who were managed expectantly, formed the study group. Women without haemoperitoneum formed the control group.

TVS was repeated after 2 and 6 months for the study group, and after 6 months for the control group, unless earlier scans were clinically indicated. These time frames were chosen based on the previous pilot study demonstrating that the median time interval between the initial visit to completion of follow-up for women in the study group was 159 days (5–6 months) and 119 days (∼4 months) for women in the control group (Bean *et al.*, 2019a). When pain and/or haemoperitoneum persisted, we followed women up until spontaneous resolution of symptoms or medical/surgical intervention was required. All women were asked to complete the ‘BSGE pelvic pain questionnaire’ at each clinic visit.

### Primary and secondary outcomes

The primary outcome of this study was the sonographically confirmed presence of deep endometriosis following the occurrence of significant acute haemoperitoneum.

Secondary outcomes were the severity of pelvic pain symptoms and health-related QOL (HR-QOL) at baseline and 6 months.

### Data collection and image acquisition

All eligible women were clinically assessed. A comprehensive demographic and clinical history was taken. Demographic variables included patient age, BMI, ethnicity, gravidity, parity, and smoking status. BMI was calculated using a calibrated scale and stadiometer in our clinic. Self-reported history of the following conditions or surgeries was recorded: Caesarean section, other abdominal surgery, autoimmune diseases, chronic fatigue syndrome, chronic pain syndrome, fibromyalgia, irritable bowel disease, irritable bowel syndrome, migraines, anxiety, and depression.

Women were offered a detailed TVS examination per routine practice, which was conducted methodically (see below). The scan was performed using a 7.5-MHz probe (Voluson E8, GE Medical Systems, Milwaukee, WI, USA).

Significant haemoperitoneum was defined as the presence of blood clots and echogenic fluid within the peritoneal cavity. Fresh blood clots are seen as thick, hyperechoic, inhomogeneous, and avascular lesions on TVS. They can be distinguished from free fluid and compressed on palpation with the transvaginal probe (Bean *et al.*, 2019a). When blood and clots were only seen in the pouch of Douglas (POD), the haemoperitoneum was classified as moderate. When blood and clots were also seen anterior to the uterus, in the uterovesical fold, it was regarded as severe haemoperitoneum (Rajah *et al.*, 2017; The ESHRE Working Group on Ectopic Pregnancy *et al.*, 2020).

Deep endometriosis was defined as lesions consisting of endometrium-like tissue, present in the abdomen, extending on or under the peritoneal surface. The lesions are typically nodular, have the ability to invade neighbouring structures, and are associated with fibrosis and anatomical distortion (International Working Group of AAGL, ESGE, ESHRE and WES *et al.*, 2021).

The systematic approach described by The IDEA Group Consensus Statement was used to examine the pelvis for signs of deep pelvic and ovarian endometriosis (Guerriero *et al.*, 2016). The uterus, adnexa, anterior, and posterior compartments were scanned to identify endometriotic nodules, hydro- and haematosalpinges, endometriotic tubo-ovarian complexes, pelvic adhesions, and ureteric dilatation. Deep endometriotic nodules were diagnosed when hypoechoic, avascular, solid lesions were seen on TVS. They could have smooth or irregular contours. They were often tender on palpation with the transvaginal probe and at the point of fixation of adjacent organs. The ovaries were then examined. Endometriomas were diagnosed when thick-walled, avascular ovarian cysts containing fluid of ‘ground-glass’ appearance were observed (Van Holsbeke *et al.*, 2010). In contrast, fresh haemorrhagic cysts typically displayed a ‘spider’s web’ appearance (Okaro and Valentin, 2004). When it was difficult to differentiate between these cyst types, TVS was conducted again 6 weeks later, by which time haemorrhagic cysts would have resolved. Pelvic adhesions and POD obliteration were assessed using the ‘sliding organs sign’ (Guerriero *et al.*, 2016) or the ‘flapping sail sign’ when filmy adhesions were present (Savelli *et al.*, 2004).

The pelvis was also searched for other gynaecological and non-gynaecological abnormalities during the ultrasound examination. Acute pathology, including acute appendicitis, acute pelvic inflammatory disease, ovarian hyperstimulation, ovarian torsion, and ureteric calculi, was identified in line with current literature (Timor-Tritsch *et al.*, 1998; Molander *et al.*, 2002; Nastri *et al.*, 2015; Bean *et al.*, 2019b; Moro *et al.*, 2020). Other abnormalities were diagnosed according to the following; adenomyosis and fibroids as described in the Morphological Uterus Sonographic Assessment (MUSA) Group Consensus Statement (Van den Bosch *et al.*, 2018), cervical and endometrial polyps as per The International Endometrial Tumour Analysis (IETA) Group Consensus Statement (Leone *et al.*, 2010) as well as more current evidence (Wong *et al.*, 2017), congenital uterine anomalies following the revised American Society for Reproductive Medicine (ASRM) classification for Müllerian anomalies (Pfeifer *et al.*, 2021), accessory cavitated uterine malformation (ACUM) and dilated pelvic veins using guidance from recent papers (Amin *et al.*, 2019; Naftalin *et al.*, 2020), and non-endometriotic ovarian cysts by pattern recognition (Valentin *et al.*, 2001).

The kidneys were examined for hydronephrosis and abnormalities, such as renal cysts, using a transabdominal 3.5-MHz ultrasound probe (Voluson E8, GE Medical Systems).

The examiners were highly experienced in the ultrasonic diagnosis of endometriosis and other gynaecological pathologies (EFSUMB Level 2) (Education and Practical Standards Committee *et al.*, 2006), having worked in a unit with a tertiary endometriosis centre for over 3 years. They were supervised by consultant gynaecologists who were all expert gynaecological ultrasound examiners (EFSUMB Level 3).

The British Society of Gynaecological Endoscopy (BSGE) questionnaire was used to measure the severity of pelvic pain symptoms and HR-QOL patients were experiencing (Byrne *et al.*, 2018) (British Society for Gynaecological Endoscopy, 2024). Pelvic pain symptoms assessed included pre-menstrual and menstrual pain, non-cyclical pelvic pain, dyspareunia, menstrual and non-menstrual dyschezia, lower back pain, dysuria, and difficulty emptying the bladder, measured on an 11-point numerical rating scale. Frequency and urgency of bowel movements, sensation of incomplete emptying, constipation, and menstrual haematochezia were graded on a 5-point Likert scale. Hormonal contraceptive usage was assessed from dichotomous data (‘yes’ and ‘no’ for each type of treatment). HR-QOL was measured using the EuroQol 5D-3L (EQ-5D-3L) questionnaire, a simple, generic tool, which is validated for clinical use and incorporated in the BSGE questionnaire (Rabin and de Charro, 2001). The EQ-5D-3L consists of two parts, one which assesses mobility, self-care, daily activities, pain and discomfort, and anxiety and depression through five questions. The EQ-5D index score is computed from the responses and ranges from 0 (death) to 1 (perfect health), The second component comprises a 100-point visual analogue scale referred to as the ‘EQ Visual Analogic Scale’ (EQ-VAS), where the user rates their overall health status, with higher scores describing better health.

All clinical and sonographic data were stored on our dedicated clinic database (Viewpoint Bildverabeitung GmbH, Munich, Germany).

### Sample size calculation and statistical analysis

Based on a previously published study (Bean *et al.*, 2019a), ∼50% of women with significant haemoperitoneum were expected to develop deep endometriosis and only 5% of women in the control group. From this, we calculated that a sample size of 30, with 15 in each group, would be required, to achieve a power of 80% and CI of 95%.

Normally distributed data was reported using mean and SD, and non-normally distributed data with median and interquartile range (IQR). Data distribution was determined by assessing skewness and kurtosis. The chi-square test or Fisher’s exact test was used to compare categorical variables between groups. Continuous variables were compared between groups using the unpaired Student’s *t*-test or Mann–Whitney test, depending on normality of the sample.

Changes in pain and QOL scores between timepoints for individuals were analysed using the paired Student’s *t*-test for normally distributed variables or the Wilcoxon matched pairs test for non-normally distributed variables. The Wilcoxon matched pairs test or the paired exact test were used to examine changes over time.

To assess change of categorical variables over time, regression methods were used, with the outcome variable being the value at 6 months, with the baseline value included as a covariate. By adjusting for the baseline value, this is akin to examining the change over time. Ordinal logistic regression was used for the ordinal outcomes, whilst logistic regression was used for the binary variables. *P*-values of <0.05 were deemed statistically significant. Statistical calculations were performed via Stata version 15.1 (StataCorp., College Station, TX, USA).

### Ethical approval

Ethical approval was obtained from the NHS Research Ethics Committee (Reference: 19/NI/0107) on 21.05.2019.

## Results

**Figure 1.**
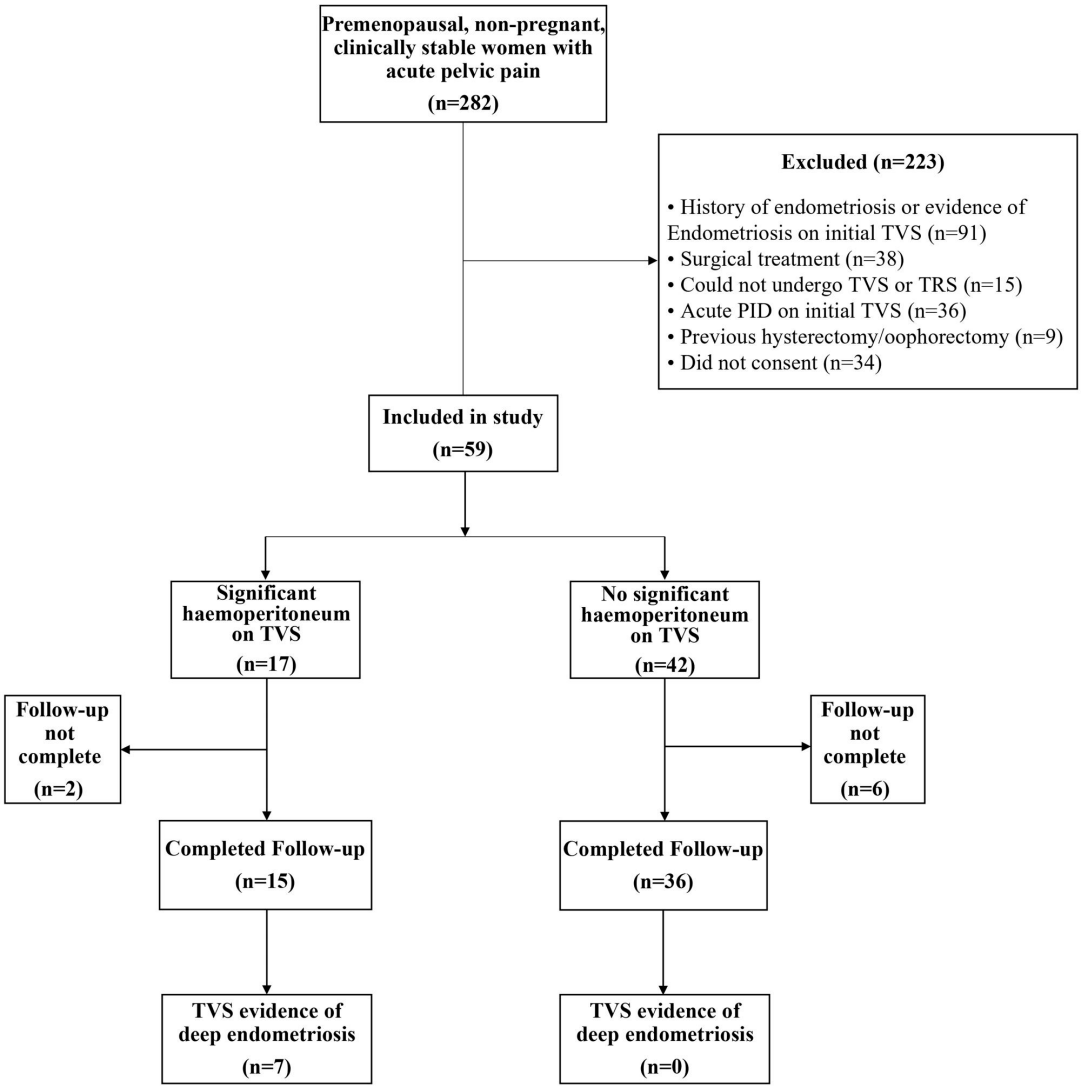
**Flowchart summarizing inclusion, exclusion, and diagnosis of women attending our acute gynaecology unit during the study period.** TVS, transvaginal ultrasound; TRS, transrectal ultrasound; PID, pelvic inflammatory disease.

During the study period, 282 eligible women presented to our clinic, of which 59 fulfilled inclusion criteria and consented to participate. Fifty-one women attended all follow-up appointments and formed the final study sample. The patient flow is presented in Fig. 1.

The demographic and clinical characteristics of the study participants are listed in Table 1, and concomitant ultrasound abnormalities in Supplementary Table S1. There were no statistically significant differences between the study and control groups at their initial visit. The participants’ analgesic use at baseline and 6 months, as well as proportions trying for pregnancy, are displayed in Supplementary Tables S2 and S3.

**Table 1.** Demographic and clinical characteristics of the study participants (N = 51), categorized by presence or absence of haemoperitoneum at initial presentation.

	**Haemoperitoneum** (N = 15), n (%)	**No haemoperitoneum** (N = 36), n (%)	*P*-value
Age (years), median (IQR)	31 (25–33)	26 (23–33)	0.24
BMI (kg/m^2^), mean ± SD	25.8 ± 5.6	24.3 ± 4.3	0.32
Ethnicity			0.33
Caucasian	9 (60.0)	23 (63.9)	
Afro-Caribbean	3 (20.0)	2 (5.6)	
South Asian	1 (6.7)	7 (19.4)	
Other	2 (13.3)	4 (11.1)	
Smoking			0.77
Non-smoker	8 (53.3)	17 (47.2)	
Ex-smoker	3 (20.0)	5 (13.9)	
Current smoker	4 (26.7)	14 (38.9)	
Gravidity			0.41
0	6 (40.0)	20 (55.6)	
1	4 (26.7)	10 (27.8)	
2+	5 (15.0)	6 (16.7)	
Parity			0.10
0	10 (66.7)	32 (88.9)	
1+	5 (33.3)	4 (11.1)	
Hormonal Contraception	3 (20)	17 (47)	0.12
Previous Caesarean section	2 (13.3)	1 (2.8)	0.2
Previous Gynaecological Surgery	3 (20.0)	3 (8.3)	0.34
Any previous Abdominal Surgery (Gynaecological or non-gynaecological)	4 (26.7)	12 (33.3)	0.75
Other Medical Conditions	5 (33.3)	4 (11.1)	0.11

No women reported a history of chronic fatigue syndrome, chronic pain syndrome, fibromyalgia, inflammatory bowel disease, or inflammatory bowel syndrome in either group. IQR, interquartile range.

Table 2 shows the primary diagnosis made on TVS at the initial presentation, including the causes for haemoperitoneum. The most common reason for haemoperitoneum was a ruptured functional haemorrhagic ovarian cyst, present in 13/15 cases (87%). The use of hormonal contraception was less frequent in women who developed deep endometriosis, but the difference was not statistically significant (47% vs 20%, *P *=* *0.1). There were 10/36 (28%) women in the ‘no haemoperitoneum’ group who were taking the combined oral contraceptive pill (COCP) or the progesterone-only pill, compared to no women in the haemoperitoneum group (*P *=* *0.02). Furthermore, all 3/15 (20%) women in the haemoperitoneum group who were using hormonal contraception, had a levonorgestrel-containing intrauterine device (Lng-IUS) *in situ*, compared to only 1/36 (2.8%) in the ‘no haemoperitoneum’ group (*P *=* *0.07).

**Table 2.** Clinical diagnosis causing acute pelvic pain in the study participants (N = 51), categorized by presence or absence of haemoperitoneum at initial presentation.

Primary diagnosis at initial TVS	**Haemoperitoneum** (N = 15), n (%)	**No haemoperitoneum** (N = 36), n (%)
Ruptured functional ovarian cyst	13 (86.7)	4 (11.1)
Recent ovulation	0 (0.0)	16 (100)
Appendicitis	0 (0.0)	2 (13.3)
Retrograde menstruation from cervical stenosis	1 (6.7)	0 (0.0)
Post-operative intra-abdominal bleeding	1 (6.7)	0 (0.0)
Haematometra	0 (0.0)	1 (2.8)
Displaced IUCD	0 (0.0)	1 (2.8)
Urinary tract infection	0 (0.0)	1 (2.8)
Ascites (secondary to CMV infection)	0 (0.0)	1 (2.8)
Unexplained pain	0 (0.0)	10 (27.8)

Where N differs from 44 women in the group who did not develop endometriosis and seven in the group who did develop endometriosis, this was due to missing data. TVS, transvaginal ultrasound; IUCD, intrauterine contraceptive device; CMV, cytomegalovirus.

After completion of follow-up, 7/15 (46.7%; 95% CI 21.3–71.4%) women with haemoperitoneum developed sonographic evidence of deep endometriosis, compared to 0/36 (0%; 95% CI 0.0–9.7%) women in the control group, who had no signs of haemoperitoneum at inclusion. Of the seven women who developed deep endometriosis, only one patient also had evidence of an ovarian endometrioma. Of the 7/15 (46.7%) women who developed deep endometriosis, 4/7 (57%; 95% CI 20.5–93.8%) presented with severe haemoperitoneum and 3/7 (43%; 95% CI 6.2–79.5%) with moderate haemoperitoneum.

**Figure 2.**
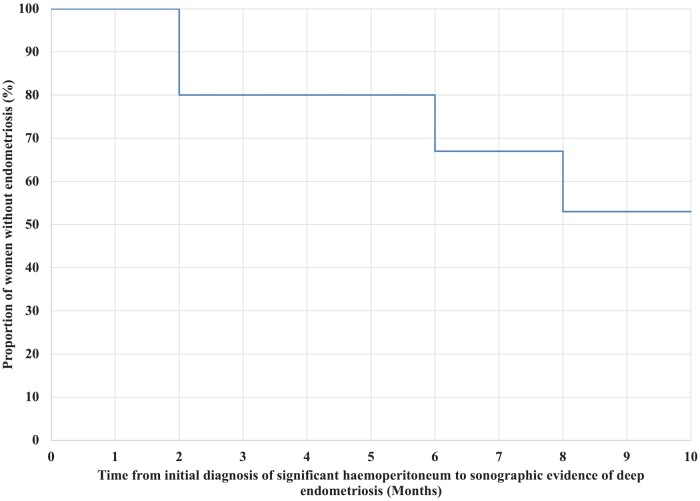
**Kaplan–Meier estimator illustrating the proportion of women who presented with acute pelvic pain and significant haemoperitoneum at initial scan, who remained free of deep endometriosis during the follow-up period.** Estimator is inclusive of 15 women.

**Figure 3.**
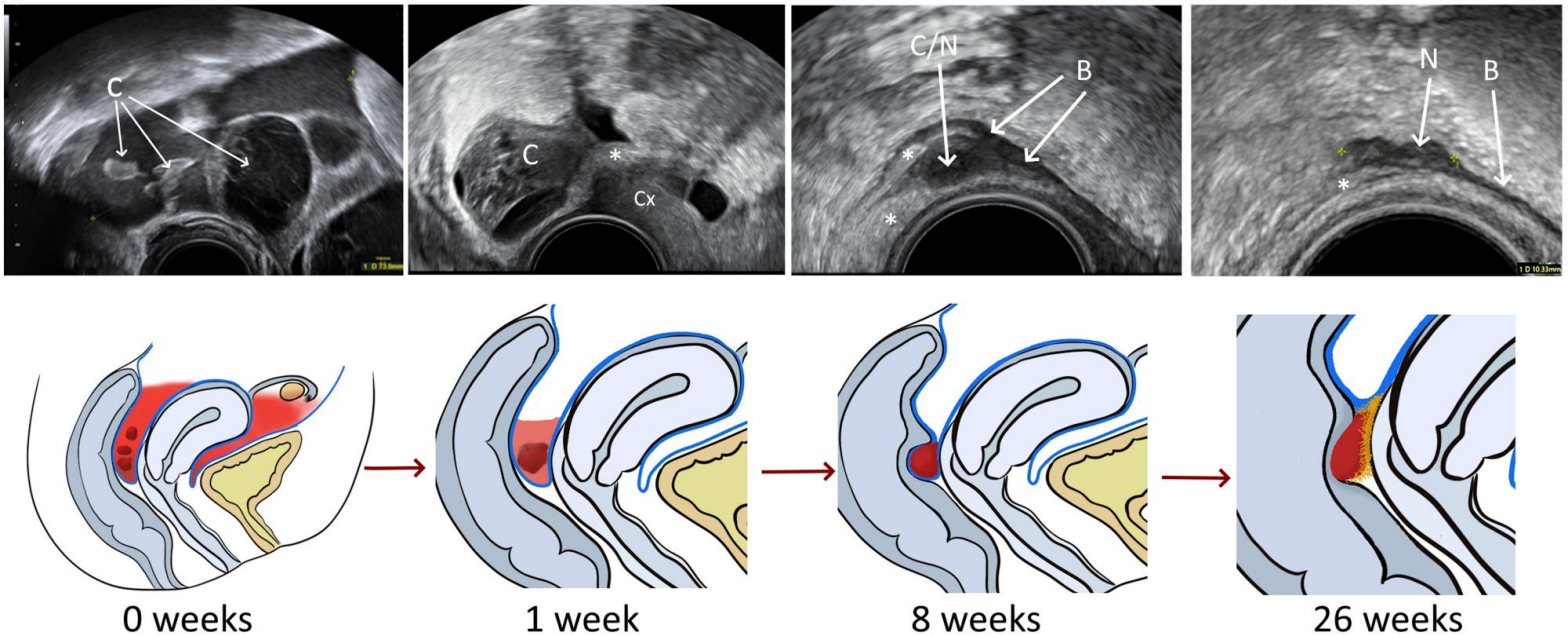
**Formation of endometriosis nodules following haemoperitoneum.** B-mode transvaginal ultrasound images in the upper row, with corresponding schematic illustrations below. 0 weeks: Significant haemoperitoneum containing smaller clots (c) secondary to a ruptured functional haemorrhagic cyst. 1 week: Free fluid in the pouch of Douglas is starting to reabsorb. A larger clot (C) is formed, located in the retrocervical region. Peritoneum shows reactive thickening (*). 8 weeks: This image illustrates the blood clot transitioning to an endometriotic nodule (C/N). The blood clot becomes smaller, more solid and hypoechoic over time, and adherent to the surrounding peritoneum and bowel. The muscularis layer of the bowel (B) appears thickened, as does the surrounding peritoneum (*). 26 weeks: The resolving clot contracts further in size and becomes a completely solid, incompressible, hypoechoic lesion (N). It is tender on palpation with the ultrasound probe and resembled the appearance of an endometriotic nodule. The nodule invades the muscularis layer of the anterior wall of the rectosigmoid colon (B). The transition of the peritoneal to a fibrotic state is visible (*). C, clot; Cx, cervix; C/N, the transitioning blood clot into an endometriotic nodule; N, endometriotic nodule; B, bowel wall.

Of the women with significant haemoperitoneum at their initial visit, the proportion who had sonographic evidence of deep endometriosis at their follow-up scans is demonstrated in Fig. 2. The formation of deep endometriosis over time is illustrated in Fig. 3. Of the 8/15 (53%) women who did not develop deep endometriosis following haemoperitoneum, the pelvic blood clots had resolved by their first follow-up visit, which occurred at 2 months in 6/8 (75%) women. In 2/8 women, this was undertaken earlier for clinical reasons, 17 and 25 days from the initial attendance.

The median number of endometriotic nodules seen per person was 1 (range 1–2) and the locations involved were the uterosacral ligaments (n = 3), retrocervical area (n = 3), rectosigmoid colon (n = 1), and bladder (n = 1). Partial obliteration of the POD was seen in 3/7 women (43%; CI 6.2–79.5%). Other pelvic adhesions involving the organs neighbouring the newly formed endometriotic nodule(s) were noted in 4/7 women (57%; CI 20.5–93.8%).

When comparing women who developed endometriosis with those who did not, there were only a few statistically significant differences in pelvic pain, bowel symptoms, and EQ-5D-3L scores (Tables 3 and 4). A greater proportion of women who later developed endometriosis reported having constipation at baseline compared to women who did not develop endometriosis (*P *=* *0.01), but this was not seen at 6 months. Women who developed endometriosis also reported greater difficulty emptying their bladder at 6 months (*P *=* *0.04) (Table 3), and they also had a lower EQ-5D index score at both baseline and 6 months (*P *=* *0.002, *P *=* *0.03, respectively) (Table 3), compared to women who did not develop endometriosis.

**Table 3.** Severity and frequency of pain symptoms and EQ-5D-3L quality of life scores in groups who did and did not develop endometriosis, at baseline and at 6 months.

Symptom	Endometriosis N = 7					No Endometriosis N = 44					Between group comparison	
	Baseline		6 months		Change in Median VAS scores 0–6 months (*P*-value) ^(**)^	Baseline		6 months		Change in Median VAS scores 0–6 months (*P*-value) ^(**)^	Baseline Median VAS scores (*P*-value)	6-month median VAS scores (*P*-value)
	n	VAS score Median [IQR]	n	VAS score Median [IQR]		n	VAS score Median [IQR]	n	VAS score Median [IQR]			
Pre-menstrual pain	6	4 [2–4]	5	2[0–8]	0.85	42	4 [1–6]	38	4[2–7]	0.52	0.72	0.58
Menstrual pain	6	6 [3–7]	5	7[2–9]	0.47	42	6 [3–8]	38	7[3–8]	0.74	0.64	0.91
Non-cyclical pain	6	5 [1–6]	6	6[0–7]	0.49	44	2 [0–5]	42	2[0–5]	0.79	0.23	0.26
Dyspareunia	6	4 [0–7]	6	2[0–5]	1.00	44	1 [0–5]	42	2[0–5]	0.99	0.48	0.84
Menstrual dyschezia	6	2 [0–8]	5	2[0–5]	0.68	43	0 [0–4]	39	0[0–3]	0.88	0.18	0.55
Non-menstrual dyschezia	6	2 [0–8]	6	3[0–5]	0.48	44	0 [0–0]	42	0[0–3]	0.37	0.10	0.27
Lower back pain	6	6 [4–8]	6	7[5–8]	0.39	44	5 [2–7]	42	5[3–7]	0.69	0.24	0.06
Bladder pain/dysuria	6	0 [0–1]	6	2[0–3]	0.09	44	0 [0–1]	42	0[0–0]	0.23	0.85	0.17
Difficulty emptying bladder	6	0 [0–0]	6	1[0–2]	0.09	44	0 [0–0]	42	0 [0–0]	0.71	0.75	**0.04**
***EQ-5D-3L***												
EQ-5D Index	6	−0.01^(*)^ [−0.07–0.19]	6	0.69^(*)^ [0.66–0.80]	**0.04**	44	0.62^(*)^ [0.24–0.73]	42	0.85^(*)^ [0.76–1.00]	**<0.001**	**0.002**	**0.03**
EQ-VAS	6	28 [15–40]	6	58 [40–85]	0.08	44	56 [35–75]	42	83 [60–91]	**<0.001**	0.09	0.11

Where N differs from 44 women in the group who did not develop endometriosis, and 7 in the group who did develop endometriosis, this was due to either ‘N/A’ being selected on the questionnaire or missing data. (*) Number representing EQ-5D Index. (**) *P*-values calculated from women who recorded scores for both timepoints for each variable only (women who recorded ‘N/A’ for either timepoint were also excluded), therefore N differed from values in table slightly in some cases. For further details, see Supplementary Table S2. VAS, Visual Analogue Scale. IQR, interquartile range. EQ-5D-3L, EuroQoL-5 Dimension-3 Level. EQ-5D Index, EuroQoL-5 Dimension Index. EQ-VAS, EuroQoL-Visual Analogue Scale.

**Table 4.** Severity and frequency of bowel symptoms scores in groups who did and did not develop endometriosis, at baseline and at 6 months.

Symptom	Category	Endometriosis N = 7					No endometriosis N = 44					Between group comparison	
		Baseline		6 months		Change in n 0–6 months (*P*-value) ^(**)^	Baseline		6 months		Change in n 0–6 months (*P*-value) ^(**)^	Baseline scores (*P*-value)	6-month scores (*P*-value)
		N	n (%)	N	n (%)		N	n (%)	N	n (%)			
Frequent Bowel Movements	Never	6	1 (17)	6	1 (17)	0.89	44	2 (5)	42	4 (10)	0.14	0.65	0.84
	Little of time		1 (17)		1 (17)			9 (20)		6 (14)			
	Some of time		0 (0)		1 (17)			11 (25)		13 (31)			
	Most of time		3 (50)		3 (50)			20 (45)		17 (40)			
	All the time		1(17)		0 (0)			2 (5)		2 (5)			
Urgent Bowel Movements	Never	6	0 (0)	6	1 (17)	0.32	44	14 (32)	42	11 (26)	0.17	0.10	0.69
	Little of time		4 (67)		3 (50)			23 (53)		19 (45)			
	Some of time		2 (33)		2 (33)			6 (14)		11 (26)			
	Most of time		0 (0)		0 (0)			1 (2)		1 (2)			
	All the time		0 (0)		0 (0)			0 (0)		0 (0)			
Sensation of incomplete bowel emptying	Never	6	0 (0)	6	0 (0)	0.78	44	16 (36)	42	12 (29)	0.43	0.47	0.79
	Little of time		5 (83)		5 (83)			14 (32)		21 (50)			
	Some of time		1 (7)		0 (7)			8 (18)		7 (17)			
	Most of time		1 (7)		1 (17)			4 (9)		1 (2)			
	All the time		0 (0)		0 (0)			2 (5)		1 (2)			
Constipation	Never	6	0 (0)	6	0 (0)	0.18	44	10 (23)	35	13 (31)	0.35	**0.01**	0.77
	Little of time		1 (17)		1 (17)			22 (50)		18 (43)			
	Some of time		4 (67)		4 (67)			9 (20)		8 (19)			
	Most of time		1 (17)		1 (17)			3 (7)		3 (7)			
	All the time		0 (0)		0 (0)			0 (0)		0 (0)			
Rectal bleeding during menstruation	Never	6	6 (100)	5	5 (100)	1.00	43	34 (79)	34	31 (76)	0.95	0.22	0.18
	Little of time		0 (0)		0 (0)			3 (7)		5 (12)			
	Some of time		0 (0)		0 (0)			4 (9)		4 (10)			
	Most of time		0 (0)		0 (0)			2 (5)		1 (2)			
	All the time		0 (0)		0 (0)			0 (0)		0 (0)			

Where N differs from 44 women in the group who did not develop endometriosis, and 7 in the group who did develop endometriosis, this was due to either ‘N/A’ being selected on the questionnaire or missing data. (*) Number representing EQ-5D Index. (**) P-values calculated from women who recorded scores for both timepoints for each variable only (women who recorded ‘N/A’ for either timepoint were also excluded), therefore N differed from values in table slightly in some cases.

We analysed the change in symptoms and QOL between baseline and 6 months (Tables 3 and 4, Supplementary Tables S4 and S5), in the women who developed endometriosis and those who did not. The only observed difference in change was EQ-5D index and EQ-VAS scores, which demonstrated that by 6 months, there was a statistically significant improvement in QOL from baseline in the group who did not develop endometriosis (*P *<* *0.001, *P *<* *0.001, respectively). In the group who did develop endometriosis, only the EQ-5D index score improved (*P *=* *0.04), not the EQ-VAS score (*P *=* *0.08) (Table 3).

The women in the group who had significant haemoperitoneum were also reviewed at 2 months. In the subgroup who developed endometriosis, EQ-5D index and EQ-VAS scores were significantly lower at initial presentation compared to the 2-month follow-up visit (*P *=* *0.03, *P *=* *0.04) (Supplementary Table S6). There were no other statistically significant changes in scores observed for pelvic pain, bowel, and urinary symptoms between baseline and 2 months, nor between 2 and 6 months (Supplementary Tables S6 and S7).

## Discussion

Our study found that nearly half of women presenting with significant acute haemoperitoneum developed deep endometriosis during follow-up, compared to none of the women without haemoperitoneum. Our primary outcome was comparable to the findings in the study by Bean *et al.* (2019a), who reported that 67% of women with haemoperitoneum developed endometriosis compared to only 3% of women without haemoperitoneum.

We observed on interval TVS examinations that in some women, blood clots did not resolve. Instead, they became more organized and solid over time, appearing more hypoechoic and smaller in size, always remaining in the same position (Fig. 3). They eventually resembled the characteristic incompressible, solid, hypoechoic appearance of endometriotic nodules, which were tender on palpation with the ultrasound probe (Guerriero *et al.*, 2016). Nodules appear different from resolving blood clots, which tend to reside in the POD, whilst endometriotic nodules directly involve the bowel wall, bladder wall, sacro-uterine ligaments, or parametria, presenting as focal abnormalities of these organs. Nodules are also different from pelvic fibrosis involving the pelvic organs and peritoneum, which typically appear hyperechoic on ultrasound scan, while the endometriotic nodules are typically hypoechoic.

It has been previously demonstrated that endometrial epithelial cell colonies are prevalent in the peritoneal fluid of 79–90% of women, regardless of the presence of endometriosis (Halme *et al.*, 1984; Kruitwagen *et al.*, 1991). As hypothesized by Bean *et al.* (2019a), when peritoneal healing occurs over a blood clot, these endometrial cells could become trapped underneath the peritoneal surface and trigger the development of deep disease. A large amount of intraperitoneal blood translates into major oxidative stress that is extremely deleterious for the delicate mesothelial cells. Such cytotoxic effect on the peritoneal lining could open the way to the extracellular matrix for these endometrial cells (Wyatt *et al.*, 2023).

Furthermore, various molecules released by activated platelets, activation of immune cells, and neuroangiogenesis in response to the haemoperitoneum might cause the endometrial cells within the clot to undergo epithelial–mesenchymal transition and fibroblast–myofibroblast transdifferentiation (Yan *et al.*, 2017). Activated platelets have also been shown to trigger endothelial to mesenchymal transition, which also occurs in endometriotic lesions (Yan *et al.*, 2020). These processes result in increased cellular proliferation, contractility, migration, invasiveness, and collagen production, ultimately leading to fibrosis (Guo, 2018; Yan *et al.*, 2020), which is a characteristic feature of endometriosis (Vigano *et al.*, 2020). This fibrosis could then lead to adhesion formation and pelvic anatomical distortion (Nisolle and Donnez, 1997), which was also observed in our study.

Blood clots were most commonly found in the posterior compartment owing to gravitational effects. This would explain why deep endometriosis and adhesions typically form in the posterior compartment, often involving the anterior bowel wall and leading to obliteration of the POD (Chapron *et al.*, 2006; Chaggar *et al.*, 2023a).

In 87% of the women who presented with acute haemoperitoneum, this was secondary to a ruptured functional haemorrhagic cyst. Patient characteristics that are positively or negatively associated with ovulation, such as frequent menstrual cycles, early menarche, parity, and oral contraceptive use, have been consistently linked to endometriosis risk (Sangi-Haghpeykar and Poindexter, 1995; Arumugam and Lim, 1997; Moen & Schei, 1997; Vercellini *et al.*, 1997). Our study offers a pathophysiological explanation for this link. This association is further supported by a recent, large cohort study, reporting a positive association between the presence of functional haemorrhagic ovarian cysts and endometriosis (Chaggar *et al.*, 2023a). Although the overall use of hormonal contraception was not statistically less frequent in women who developed deep endometriosis (47% vs 20%, *P *=* *0.1), given the large difference in the two figures, a type II error may have occurred here. This is likely owing to the small sample size. Additionally, there was a trend towards more women using the Lng-IUS in the haemoperitoneum group compared to the ‘no haemoperitoneum’ group (*P *=* *0.07). This is less likely to inhibit ovulation than the COCP or oral progestogens, which were more commonly being used in the ‘no haemoperitoneum’ group (*P *=* *0.02).

Why not all women with blood clots in the pelvis develop deep endometriosis is uncertain. Kapczuk *et al.* (2023) reported that only 46% women who had developed retrograde menstruation caused by obstructive uterine anomalies were diagnosed with endometriosis at surgery, which is very similar to our findings. The factors which determine the severity of inflammatory response to the presence of blood within the peritoneal cavity are currently unknown and this requires further research. Of the 8/15 (53%) women who did not develop deep endometriosis following haemoperitoneum, the pelvic blood clots had resolved within 2 months. In contrast, in 5/7 (71%) women who developed deep endometriosis, it took at least 6 months for the blood clots to completely resolve (Fig. 2). Alternative explanations could involve the role of genetic predisposition, inflammatory changes, and individual immunological factors (Burney and Giudice, 2012; Zubrzycka *et al.*, 2020). Our sample size did not allow us to establish a reliable correlation between demographic and clinical covariates and the risk of developing haemoperitoneum or endometriosis.

We documented clinical symptoms and HR-QOL in order to investigate the potential clinical significance of *de novo* formation of deep endometriosis nodules. Observed differences, such as a higher prevalence of constipation at baseline and difficulties emptying the bladder at 6 months, in the endometriosis group do not seem clinically relevant given the small size of the endometriosis group.

We observed a statistically significant increase in the EQ-5D scores between baseline and 6 months for the group who developed endometriosis, but not for the EQ-VAS, unlike the ‘no endometriosis’ group, which noted improvements in both QOL variables. The improvements are likely linked to baseline data being obtained while the participants were admitted with acute pain. Although it did not quite reach statistical significance, the EQ-VAS did also show some improvement in the endometriosis group (*P = *0.08). These findings could have also been influenced by the small sample size and the length of the follow-up period, which was probably too short to see a potential clinical effect of endometriosis, which is not always symptomatic. The median EQ-5D index scores at 6 months were 0.73 and 0.85 for the endometriosis and ‘no endometriosis’ groups, respectively, both of which are considered clinically good.

Our findings suggest that because women with significant haemoperitoneum are more likely to develop deep endometriosis, surgical management (laparoscopy and washout) could be offered as a preventative measure, even if they are clinically stable.

The strengths of this study include the innovative hypothesis, the prospective design and a high quality of ultrasound examination with clearly defined diagnostic criteria. Although this was not a single-operator study, which would theoretically reduce inter-observer variability, all examiners were extensively trained in the ultrasound diagnosis of deep endometriosis. They belonged to the same academic group, and were using the same model of ultrasound machines and transvaginal probes, allowing for a consistent approach to examinations. Furthermore, recent studies have demonstrated high inter-observer reproducibility in the detection of deep endometriotic nodules (Bean *et al.*, 2020; Chaggar *et al.*, 2023b).

This study was designed as validation of our previous research and was able to show that the findings from the initial pilot study are reproducible. The pilot study provided valuable insights that allowed us to refine the design of our current study, perform a more accurate sample size calculation, and establish a more standardized follow-up protocol. Additionally, we were able to monitor participants’ pain scores over time, providing further insight into the evolution of these scores over the study period. Our study also demonstrated a strong relationship between haemorrhagic cysts and the development of deep endometriosis, as well as the potential protective nature of oral contraceptive pills.

Several limitations of this study need to be acknowledged. Firstly, it cannot be said for certain whether minimal, superficial endometriosis was already present at the start of the study and how this could have contributed to the development of deep endometriosis. However, this limitation also applies to women without haemoperitoneum, which was the only identifiable difference between the groups who did and did not develop deep endometriosis. Furthermore, while the ultrasound findings were in line with deep endometriosis, we have no histological data to prove that they in fact represent endometriosis. This should be investigated in future studies.

While we have investigated changes in HR-QOL and symptom scores, without finding clinically significant differences over time and between groups, the baseline score was taken when the patient was admitted with acute pain. This could likely have influenced the baseline towards a lower score.

Although the sample size in this study was calculated using findings from a previous similar study, it was still a small sample and larger studies would be required to draw definitive conclusions, particularly regarding analysis of consequences of the newly formed endometriotic lesions on fertility. Only a small proportion of our cohort was trying to conceive, preventing us from reaching sufficient conclusions regarding fertility.

Furthermore, one of the most common causes of significant haemoperitoneum in pregnant patients is ruptured ectopic pregnancy, which cannot safely be managed expectantly. It is possible that the haemoperitoneum would have behaved in the same way in pregnant women, leading to the development of deep endometriosis. However, the morphology and behaviour of endometriosis in ongoing pregnancies are very different compared to non-pregnant women (Bean *et al.*, 2023) and a separate study would be necessary to look at this.

In addition, although none of the women recruited to our study had history of recent egg collection, this is another important cause of significant haemoperitoneum. In fact, the previous preliminary study (Bean *et al.*, 2019a) did report that two of their cases of haemoperitoneum were secondary to this.

In conclusion, our study provides further evidence to suggest that significant haemoperitoneum may be a precursor of deep endometriosis in some women. Clinically stable women presenting with acute pelvic pain and significant haemoperitoneum should be counselled about the risk of developing deep endometriosis and offered expectant or surgical management. However, larger future studies need to be conducted to assess women over a longer period of time to see whether the effect on pain and QOL worsens over time. Fertility implications should also be assessed in more detail, as well as more confounding factors adjusted for in the QOL analysis. Suppression of ovulation and formation of functional cysts, e.g. with combined and progesterone-only contraceptive pills, should be investigated for the prevention of significant haemoperitoneum and development of deep endometriosis.

## Supplementary Material

hoae036_Supplementary_Data

## Data Availability

The data underlying this article are available in the article and in its online supplementary material.
