# Supplementary material for: Development of deep pelvic endometriosis following acute haemoperitoneum: a prospective ultrasound study
Source: Hum Reprod Open. 2024 May 29;2024(3):hoae036. doi: 10.1093/hropen/hoae036 (PMC11189661; doi:10.1093/hropen/hoae036)
Supplement: hoae036_Supplementary_Data [file hoae036_supplementary_data.docx]

**Supplementary Table S1:** Presence of concomitant diagnoses in the study participants (N=51), categorised by the presence or absence of hemoperitoneum at initial presentation.

| **Variable** | **Haemoperitoneum**  (N=15), n (%) | **No Haemoperitoneum**  (N=36), n (%) | ***P*-value** |
| --- | --- | --- | --- |
| Adenomyosis | 1 (6.7) | 3 (8.3) | 1.0 |
| Uterine fibroids | 2 (13.3) | 4 (11.1) | 1.0 |
| Congenital uterine anomalies | 0 (0.0) | 1 (2.8) | 1.0 |
| Hydrosalpinges/Haematosalpinges/  Tubo-ovarian abscess | 1 (6.7) | 1 (2.8) | 0.5 |
| Non-endometriotic, non-functional ovarian cysts | 2 (13.3) | 2 (5.6) | 0.6 |
| Para-ovarian cysts | 0 (0.0) | 5 (13.9) | 0.3 |

^Accessory cavitated uterine malformation and dilated pelvic veins were not seen on transvaginal ultrasound in any of the women in our cohort.^

**Supplementary Table S2:** Analgesia use and length of time trying for pregnancy between groups who did and did not develop endometriosis, at baseline and 6 months.

| **VARIABLE** | **ENDOMETRIOSIS** | | | | **NO ENDOMETRIOSIS** | | | | **BETWEEN GROUP CHANGE** | |
| --- | --- | --- | --- | --- | --- | --- | --- | --- | --- | --- |
|  | **Baseline** | | **6 months** | | **Baseline** | | **6 months** | | **Baseline (*P*-Value)** | **6-month (*P*-Value)** |
|  | **N** | **n (%)** | **N** | **n (%)** | **N** | **n (%)** | **N** | **n (%)** |  |  |
| **Analgesia use** | | | | | | | | | | |
| Paracetamol | 6/7 | 4 (67) | 6/7 | 4 (67) | 42/44 | 41 (93) | 42/44 | 4 (67) | 0.10 | 0.61 |
| NSAIDs | 6/7 | 3 (50) | 6/7 | 3 (50) | 42/44 | 33 (75) | 42/44 | 1 (17) | 0.33 | **0.02** |
| Opiates | 6/7 | 1 (17) | 6/7 | 1 (17) | 42/44 | 4 (9) | 42/44 | 1 (17) | 0.49 | 0.43 |
| **Fertility** | | | | | | | | | | |
| Trying for pregnancy | 6/7 | 0 (0) | 6/7 | 0 (0) | 44/44 | 5 (12) | 39/44 | 0 (0) | 1.00 | 1.00 |
| Trying for pregnancy >18 months | 0/7 | - | 0/7 | - | 5/44 | 1 (20) | - | - | - | - |

^Where N differs from 44 women in group who did not develop endometriosis and 7 in the group who did develop endometriosis, this was due to either ‘N/A’ being selected on the questionnaire or missing data. NSAIDs, non-steroidal anti-inflammatories.^

**Supplementary Table S3:** Change in use of analgesia and length of time trying for pregnancy from baseline to 6 months, within each group, and between groups who did and did not develop endometriosis (difference in change between groups represented by *P*-value in final column).

| **SYMPTOMS** | **ENDOMETRIOSIS** | | | | | **NO ENDOMETRIOSIS** | | | | | **BETWEEN GROUP COMPARISON**  **(*P*-Value)** |
| --- | --- | --- | --- | --- | --- | --- | --- | --- | --- | --- | --- |
|  | **N** | **Baseline** | **6 months** | **Change 0-6 months**  **% (95% CI)** | ***P*-Value** | **N** | **Baseline** | **6 months** | **Change 0-6 months**  **% (95% CI)** | ***P*-Value** |  |
|  |  | **n (%)** | **n (%)** |  |  |  | **n (%)** | **n (%)** |  |  |  |
| **Analgesia Use** | | | | | | | | | | | |
| Paracetamol | 5/7 | 3 (60) | 4 (80) | 20 (-35–75) | 1.00 | 42/44 | 39 (93) | 33 (79) | -14 (-27– -1) | **0.03** | 0.99 |
| Ibuprofen | 5/7 | 2 (40) | 1 (20) | -20 (-56–23) | 1.00 | 42/44 | 31 (74) | 29 (69) | -5 (-16–7) | 0.63 | 0.12 |
| Opiates | 5/7 | 0 (0) | 0 (0) | 0 (-20– 20) | 1.00 | 42/44 | 4 (10) | 3 (7) | -2 (-15–10) | 1.00 | 0.44 |
| **Fertility** | | | | | | | | | | | |
| Trying for pregnancy | 5/7 | 0 (20) | 1 (20) | 20 (-35–75) | 1.00 | 42/44 | 5 (12) | 7 (17) | 5 (-7–16) | 0.63 | 0.41 |

^Where N differs from 44 women in the group who did not develop endometriosis and 7 in the group who did develop endometriosis, this was due to missing data.CI, confidence interval. NSAIDs, non-steroidal anti-inflammatories.^

**Supplementary Table S4:** Severity and frequency of pain symptoms and EQ-5D-3L quality of life scores in groups who did and did not develop endometriosis, at baseline and at 6 months.

| **SYMPTOMS** | **ENDOMETRIOSIS**  **N= 7** | | | | **NO ENDOMETRIOSIS**  **N= 44** | | | | **BETWEEN GROUP COMPARISON** |
| --- | --- | --- | --- | --- | --- | --- | --- | --- | --- |
|  |  | **Baseline** | **6 months** | **Change in Median VAS scores 0-6 months (*P*- value)** |  | **Baseline** | **6 months** | **Change in Median VAS scores 0-6 months (*P*- value)** | **Change in Median VAS scores 0-6 months (*P*- value)** |
|  | **n** | **VAS score**  **Median [IQR]** | **VAS score**  **Median [IQR]** |  | **n** | **VAS score**  **Median [IQR]** | **VAS score**  **Median**  **[IQR]** |  |  |
| Pre-menstrual pain | 4 | 3 [1–4] | 1 [0–7] | 0.85 | 37 | 4 [1–7] | 4[2–7] | 0.52 | 0.61 |
| Menstrual pain | 4 | 4 [1–6] | 5 [1–9] | 0.47 | 37 | 6 [4–8] | 7[3–8] | 0.74 | 0.26 |
| Non-cyclical pain | 5 | 3 [1–6] | 5 [0–7] | 0.49 | 42 | 3 [0–5] | 2[0–5] | 0.79 | 0.50 |
| Dyspareunia | 5 | 0 [0–7] | 2 [0–6] | 1.00 | 42 | 1 [0–5] | 2[0–5] | 0.99 | 1.00 |
| Menstrual dyschezia | 5 | 1 [0–5] | 4 [0–6] | 0.68 | 38 | 0 [0–4] | 0[0–3] | 0.88 | 0.82 |
| Non-menstrual dyschezia | 5 | 0 [0–6] | 4 [0–6] | 0.48 | 42 | 0 [0–0] | 0[0–3] | 0.37 | 0.69 |
| Lower back pain | 5 | 5 [4–8] | 7 [5–8] | 0.39 | 42 | 2 [2–7] | 5[3–7] | 0.69 | 0.60 |
| Bladder pain/dysuria | 5 | 0 [0–1] | 3 [0–5] | 0.09 | 42 | 0 [0–2] | 0[0–0] | 0.23 | **0.03** |
| Difficulty emptying bladder | 5 | 0 [0–0] | 1 [0–5] | 0.09 | 42 | 0 [0–0] | 0 [0–0] | 0.23 | **0.004** |
| ***EQ-5D-3L*** |  |  |  |  |  |  |  |  |  |
| EQ-5D Index ^(*)^ | 5 | 0.00 [-0.07–0.28] | 0.73 [0.66–0.90] | **0.04** | 42 | 0.62 [0.26–0.73] | 0.85 [0.70–1.00] | **<0.001** | **0.02** |
| EQ-VAS | 5 | 40 [15–60] | 70 [38–88] | 0.08 | 42 | 56 [35–75] | 83 [60–92] | **<0.001** | 0.76 |

^Where N differs from 44 women in the group who did not develop endometriosis and 7 in the group who did develop endometriosis, this was due to either ‘N/A’ being selected on the questionnaire or missing data. (*) Number representing EQ-5D Index rather than VAS score. IQR, interquartile range. EQ-5D-3L, EuroQoL-5 Dimension-3 Level. EQ-5D Index, EuroQoL-5 Dimension Index. EQ-VAS, EuroQoL-Visual Analogue Scale.^

**Supplementary Table S5:** Frequency of bowel symptoms in groups who did and did not develop endometriosis, at baseline and at 6 months.

| **SYMPTOM** | **CATEGORY** | **ENDOMETRIOSIS**  **N=7** | | | | **NO ENDOMETRIOSIS**  **N= 44** | | | | **BETWEEN GROUP COMPARISON** |
| --- | --- | --- | --- | --- | --- | --- | --- | --- | --- | --- |
|  |  | **N** | **Baseline**  **n (%)** | **6 months**  **n (%)** | **Change in n 0-6 months (*P*- value)** | **N** | **Baseline**  **n (%)** | **6 months**  **n (%)** | **Change in n 0-6 months (*P*- value)** | **Change in n**  **0-6 months**  **(*P*-Value)** |
| Frequent Bowel Movements | Never | 5 | 1 (20) | 1 (20) | 0.89 | 42 | 0 (0) | 4 (10) | 0.14 | 0.75 |
|  | Little of time |  | 1 (20) | 1 (20) |  |  | 9 (21) | 6 (14) |  |  |
|  | Some of time |  | 0 (0) | 0 (0) |  |  | 11 (26) | 13 (31) |  |  |
|  | Most of time |  | 2 (40) | 3 (60) |  |  | 20 (48) | 17 (40) |  |  |
|  | All the time |  | 1(20) | 0 (0) |  |  | 2 (5) | 2 (5) |  |  |
| Urgent Bowel Movements | Never | 5 | 0 (0) | 1 (20) | 0.32 | 42 | 12 (29) | 11 (26) | 0.17 | 0.45 |
|  | Little of time |  | 3 (60) | 3 (60) |  |  | 23 (55) | 19 (45) |  |  |
|  | Some of time |  | 2 (40) | 1 (20) |  |  | 6 (14) | 11 (26) |  |  |
|  | Most of time |  | 0 (0) | 0 (0) |  |  | 1 (2) | 1 (2) |  |  |
|  | All the time |  | 0 (0) | 0 (0) |  |  | 0 (0) | 0 (0) |  |  |
| Sensation of incomplete bowel emptying | Never | 5 | 0 (0) | 0 (0) | 0.78 | 42 | 15 (36) | 12 (29) | 0.43 | 0.68 |
|  | Little of time |  | 5 (100) | 5 (83) |  |  | 14 (33) | 21 (50) |  |  |
|  | Some of time |  | 0 (0) | 0 (0) |  |  | 7 (17) | 7 (17) |  |  |
|  | Most of time |  | 0 (0) | 0 (0) |  |  | 4 (10) | 1 (2) |  |  |
|  | All the time |  | 0 (0) | 0 (0) |  |  | 2 (5) | 1 (2) |  |  |
| Constipation | Never | 5 | 0 (0) | 0 (0) | 0.18 | 42 | 10 (24) | 13 (31) | 0.35 | 0.54 |
|  | Little of time |  | 1 (20) | 3 (60) |  |  | 20 (48) | 18 (43) |  |  |
|  | Some of time |  | 4 (80) | 0 (0) |  |  | 9 (21) | 8 (19) |  |  |
|  | Most of time |  | 0 (0) | 1 (20) |  |  | 3 (7) | 3 (7) |  |  |
|  | All the time |  | 0 (0) | 0 (0) |  |  | 0 (0) | 0 (0) |  |  |
| Rectal bleeding during menstruation | Never | 5 | 5 (100) | 5 (100) | 1.00 | 40 | 33 (83) | 30 (75) | 0.95 | 0.18 |
|  | Little of time |  | 0 (0) | 0 (0) |  |  | 2 (5) | 5 (13) |  |  |
|  | Some of time |  | 0 (0) | 0 (0) |  |  | 3 (8) | 4 (10) |  |  |
|  | Most of time |  | 0 (0) | 0 (0) |  |  | 2 (5) | 1 (3) |  |  |
|  | All the time |  | 0 (0) | 0 (0) |  |  | 0 (0) | 0 (0) |  |  |

^Where N differs from 44 women in the group who did not develop endometriosis and 7 in the group who did develop endometriosis, this was due to either ‘N/A’ being selected on the questionnaire or missing data.^

**Supplementary Table S6:** Change in severity of pelvic pain and urinary symptoms, and EQ-5D-3L scores, from baseline to 2 months, and from 2 to 6 months, in the group who developed endometriosis.

| **Pain symptom** | **Baseline**  **Median VAS score [IQR]** | **2 months**  **Median VAS score [IQR]** | **N** | **Change in median VAS score, 0-2 months**  **Median (95% CI)** | ***P*-value** | **2 months**  **Median VAS score [IQR]** | **6 months**  **Median VAS score [IQR]** | **N** | **Change in median VAS score, 2-6 months**  **Median (95% CI)** | ***P*-value** |
| --- | --- | --- | --- | --- | --- | --- | --- | --- | --- | --- |
| Pre-menstrual pain | 3 [1­–7] | 6 [2–8] | 5/7 | 2 (-1–4) | 0.17 | 5 [1–8] | 1 [0–7] | 4/7 | -2 (-4–0) | 0.16 |
| Menstrual pain | 4 [1–6] | 5 [3–7] | 4/7 | 1 (-1–4) | 0.35 | 5 [3–7] | 5 [1–9] | 4/7 | -2 (-2–2) | 0.71 |
| Non-cyclical pain | 5 [1–7] | 4 [2–8] | 6/7 | 1 (-5–6) | 0.09 | 4 [2–8] | 5 [0–7] | 5/7 | 0 (-4–3) | 0.48 |
| Dyspareunia | 0 [0–7] | 3 [0–9] | 5/7 | 1 (0–3) | 0.09 | 3 [0–9] | 2 [0–6] | 5/7 | -1 (-4–2) | 0.28 |
| Menstrual dyschezia | 1 [0–6] | 4 [0–8] | 4/7 | 0 (-1–7) | 0.84 | 4 [0–8] | 5 [1–6] | 4/7 | -1 (-2–2) | 0.85 |
| Non-menstrual dyschezia | 2 [0–8] | 0 [0–2] | 6/7 | 0 (0–8) | 0.16 | 0 [0–4] | 5 [0–6] | 5/7 | 0 (-2–5) | 0.39 |
| Lower back pain | 6 [4­–8] | 6 [6–8] | 6/7 | 1 (-3–3) | 0.52 | 6 [6–8] | 7 [5–8] | 5/7 | -1 (-1–1) | 0.32 |
| Bladder pain / dysuria | 0 [0–3] | 2 [0–7] | 6/7 | 2 (-8–8) | 0.52 | 4 [0–7] | 3 [0–5] | 5/7 | -1 (-6–7) | 0.59 |
| Difficulty emptying bladder | 0 [0–2] | 0 [0–2] | 6/7 | 0 (-6–6) | 1.00 | 0 [0–4] | 1 [0–5] | 5/7 | 0 (0–2) | 0.16 |
| ***EQ-5D-3L*** |  |  |  |  |  |  |  |  |  |  |
| EQ5D Index ^(*)^ | -0.01 [  -0.07–0.23] | 0.80 [0.52–1.00] | 6/7 | 0.72 (0.36–1.05) | **0.03** | 0.81 [0.52–1.00] | 0.73 [0.66–0.90] | 5/7 | 0.00 (-0.20–0.14) | 1.00 |
| EQ-VAS | 28 [15–50] | 85 [73–94] | 6/7 | 45 (4–80) | **0.04** | 80 [65–92] | 70 [38–88] | 5/7 | -10 (-53–10) | 0.28 |

^Where N differs from 7 women, this was due to either ‘N/A’ being selected on the questionnaire or missing data. (*) Number representing EQ-5D Index. IQR, interquartile group. EQ-5D-3L, EuroQoL-5 Dimension-3 Level. EQ-5D Index, EuroQoL-5 Dimension Index. EQ-VAS, EuroQoL-Visual Analogue Scale.^

**Supplementary Table S7:** Change in frequency of bowel symptoms, from baseline to 2 months, and from 2 to 6 months, in the group who developed endometriosis.

| **Bowel Symptom** | **Baseline**  **n (%)** | **2 months**  **n (%)** | **N** | **Change in n,**  **0-2 months**  ***P*-value** | **2 months**  **n (%)** | **6 months**  **n (%)** | **N** | **Change in n,**  **2-6 months**  ***P*-value** |
| --- | --- | --- | --- | --- | --- | --- | --- | --- |
| **Frequent bowel movements** | | | | | | | | |
| Never | 1 (17) | 0 (0) | 6/7 | 0.49 | 0 (0) | 1 (20) | 5/7 | 0.48 |
| Little of the time | 1 (17) | 1 (17) |  |  | 1 (20) | 1 (20) |  |  |
| Some of the time | 0 (0) | 0 (0) |  |  | 0 (0) | 0 (0) |  |  |
| Most of the time | 3 (50) | 4 (67) |  |  | 4 (80) | 3 (60) |  |  |
| All of the time | 1 (17) | 1 (17) |  |  | 0 (0) | 0 (0) |  |  |
| **Urgent bowel movements** | | | | | | | | |
| Never | 0 (0) | 1 (17) | 6/7 | 0.73 | 1 (20%) | 1 (20%) | 5/7 | 0.67 |
| Little of the time | 4 (67) | 2 (33) |  |  | 1 (40%) | 3 (60%) |  |  |
| Some of the time | 2 (33) | 3 (50) |  |  | 2 (40%) | 1 (20%) |  |  |
| Most of the time | 0 (0) | 0 (0) |  |  | 0 (0%) | 0 (0%) |  |  |
| All of the time | 0 (0) | 0 (0) |  |  | 0 (0%) | 0 (0%) |  |  |
| **Sensation of incomplete emptying** | | | | | | | | |
| Never | 0 (0) | 0 (0) | 6/7 | 0.28 | 0 (0) | 2 (40) | 5/7 | 0.16 |
| Little of the time | 5 (83) | 2 (33) |  |  | 2 (40) | 1 (20) |  |  |
| Some of the time | 0 (0) | 3 (50) |  |  | 2 (40) | 1 (20) |  |  |
| Most of the time | 1 (17) | 1 (17) |  |  | 1 (20) | 1 (20) |  |  |
| All of the time | 0 (0) | 0 (0) |  |  | 0 (0) | 0 (0) |  |  |
| **Constipation** | | | | | | | | |
| Never | 0 (0) | 0 (0) | 6/7 | 0.74 | 0 (0) | 1 (20) | 5/7 | 0.16 |
| Little of the time | 1 (17) | 2 (33) |  |  | 2 (40) | 3 (60) |  |  |
| Some of the time | 4 (67) | 2 (33) |  |  | 1 (20) | 0 (0) |  |  |
| Most of the time | 1 (17) | 1 (17) |  |  | 1 (20) | 1 (20) |  |  |
| All of the time | 0 (0) | 1 (17) |  |  | 1 (20) | 0 (0) |  |  |
| **Rectal bleeding during menstruation** | | | | | | | | |
| Never | 4 (100) | 4 (100) | 4/7 | 1.00 | 4 (100) | 4 (100) | 4/7 | 1.00 |
| Little of the time | 0 (0) | 0 (0) |  |  | 0 (0) | 0 (0) |  |  |
| Some of the time | 0 (0) | 0 (0) |  |  | 0 (0) | 0 (0) |  |  |
| Most of the time | 0 (0) | 0 (0) |  |  | 0 (0) | 0 (0) |  |  |
| All of the time | 0 (0) | 0 (0) |  |  | 0 (0) | 0 (0) |  |  |

^Where N differs from 7 women this was due to either ‘N/A’ being selected on the questionnaire or missing data.^
